# Risk of metachronous contralateral breast cancer in patients with primary invasive lobular breast cancer: Results from a nationwide cohort

**DOI:** 10.1002/cam4.5235

**Published:** 2022-09-20

**Authors:** Delal Akdeniz, Iris Kramer, Carolien H. M. van Deurzen, Bernadette A. M. Heemskerk‐Gerritsen, Michael Schaapveld, Pieter J. Westenend, Adri C. Voogd, Agnes Jager, Ewout W. Steyerberg, Stefan Sleijfer, Marjanka K. Schmidt, Maartje J. Hooning

**Affiliations:** ^1^ Department of Medical Oncology Erasmus MC Cancer Institute Rotterdam the Netherlands; ^2^ Division of Psychosocial Research and Epidemiology Netherlands Cancer Institute Amsterdam the Netherlands; ^3^ Division of Molecular Pathology Netherlands Cancer Institute Amsterdam the Netherlands; ^4^ Department of Pathology Erasmus MC Rotterdam the Netherlands; ^5^ Department of Pathology Albert Schweitzer Hospital Dordrecht the Netherlands; ^6^ Department of Research and Development Netherlands Comprehensive Cancer Organization (IKNL) Utrecht the Netherlands; ^7^ Department of Epidemiology Maastricht University Maastricht the Netherlands; ^8^ Department of Public Health Erasmus MC Rotterdam the Netherlands; ^9^ Department of Biomedical Data Sciences Leiden University Medical Centre Leiden the Netherlands

**Keywords:** contralateral breast cancer, lobular histology, metachronous, risk factor

## Abstract

Lobular primary breast cancer (PBC) histology has been proposed as a risk factor for contralateral breast cancer (CBC), but results have been inconsistent. We investigated CBC risk and the impact of systemic therapy in lobular versus ductal PBC. Further, CBC characteristics following these histologic subtypes were explored. We selected 74,373 women diagnosed between 2003 and 2010 with stage I‐III invasive PBC from the nationwide Netherlands Cancer Registry. We assessed absolute risk of CBC taking into account competing risks among those with lobular (*n* = 8903), lobular mixed with other types (*n* = 3512), versus ductal (*n* = 62,230) histology. Hazard ratios (HR) for CBC were estimated in a cause‐specific Cox model, adjusting for age at PBC diagnosis, radiotherapy, chemotherapy and/or endocrine therapy. Multivariable HRs for CBC were 1.18 (95% CI: 1.04–1.33) for lobular and 1.37 (95% CI: 1.16–1.63) for lobular mixed versus ductal PBC. Ten‐year cumulative CBC incidences in patients with lobular, lobular mixed versus ductal PBC were 3.2%, 3.6% versus 2.8% when treated with systemic therapy and 6.6%, 7.7% versus 5.6% in patients without systemic therapy, respectively. Metachronous CBCs were diagnosed in a less favourable stage in 19%, 26% and 23% and less favourable differentiation grade in 22%, 33% and 27% than the PBCs of patients with lobular, lobular mixed and ductal PBC, respectively. In conclusion, lobular and lobular mixed PBC histology are associated with modestly increased CBC risk. Personalised CBC risk assessment needs to consider PBC histology, including systemic treatment administration. The impact on prognosis of CBCs with unfavourable characteristics warrants further evaluation.

## Graphical abstract

In a nationwide cohort of 74,373 primary breast cancer (PBC) patients, patients with lobular and especially those with lobular mixed histology had higher contralateral breast cancer (CBC) risks as compared to patients with ductal histology. Systemic treatment was associated with decreased CBC risk in all patients. The results of this study contribute to personalized risk assessment, with potential impact on treatment and follow‐up decisions for PBC.

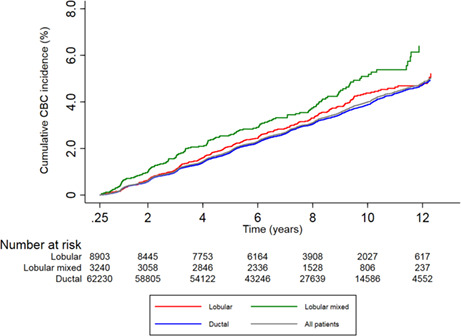

## 1 INTRODUCTION

Advances in treatment and in screening methods have led to improved breast cancer survival over the last 2–3 decades.^1^ Estimating and preventing long‐term risks, in particular the risk of contralateral breast cancer (CBC), have therefore become more relevant. Identifying risk factors for CBC can contribute to personalised risk management.

Around 10–15% of primary breast cancers (PBC) have a lobular histology. Lobular PBC histology has been proposed as a risk factor for CBC but results of previous studies have been inconsistent.^2^, ^3^, ^4^, ^5^, ^6^, ^7^ Differences in study design (i.e. small and heterogeneous study populations) could explain this inconsistency. Also, part of the lobular PBCs have a mixed or a mixed non‐classic type lobular histology rather than a classic type, but have not always been analysed as a separate group. Especially the mixed non‐classic types seem to entail a different entity.^8^ Also, the increased use of neo‐adjuvant and adjuvant systemic treatment in more recent years may have led to a decrease in CBC risk following a lobular PBC, but this warrants further investigation.

Further, little is known about the tumour characteristics of CBC following a lobular or lobular mixed PBC. If we can distinguish lobular or lobular mixed subtypes that are associated with more aggressive types of CBC, these patients might benefit from earlier detection.

In a large nation‐wide cohort, we aimed to assess metachronous CBC risk in patients with lobular or lobular mixed type PBC as compared to ductal PBC. In addition, we assessed the associations with systemic treatment (i.e. chemotherapy and/or endocrine therapy) and compared characteristics of CBCs following a lobular, lobular mixed or ductal PBC.

## 2 METHODS

### 2.1 Data collection

From the Netherlands Cancer Registry (NCR), we requested all patient, tumour, treatment and follow‐up data from women, diagnosed with invasive PBC at age ≥ 18 years between 2003 and 2010, without a previous diagnosis of invasive cancer (except basal cell cancer or squamous cell cancer of the skin). The NCR receives notifications of all new malignancies from the Dutch nationwide network and registry of histopathology and cytopathology (PALGA), and from the national hospital discharge databank containing the discharge diagnosis of all patients from Dutch hospitals. Trained research assistants collect patient, tumour and treatment information from pathology reports and medical files within the hospitals. In addition, vital status is assessed yearly by linkage with the Nationwide Municipal Administrative Database. The review boards of the NCR and PALGA approved the proposal and the data were handled in accordance to the privacy regulations for medical research.^9^ All data were anonymous to the researchers involved.

**FIGURE 1:**
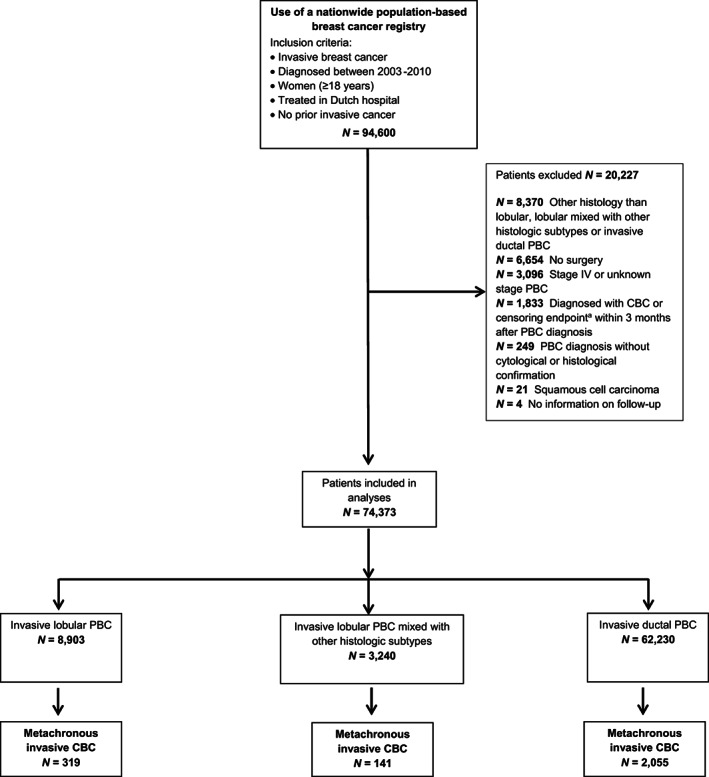
Overview of the cohort. CBC, contralateral breast cancer; PBC, primary breast cancer; ^a^endpoints: second breast or invasive non‐breast tumour (except non‐melanoma skin cancer); DCIS ipsilateral or contralateral; death; end of follow‐up (31/12/2015).

Of the 94,600 potentially eligible patients, we selected all women diagnosed with pathologically confirmed stage I‐III PBC, having either lobular histology, lobular histology mixed with other subtypes (mainly mixed with ductal histology [92%]; from now on referred to as “lobular mixed”) or ductal histology (Figure 1).

### 2.2 Statistical analysis

We compared the lobular and lobular mixed histologic groups with ductal using chi‐square statistics for categorical characteristics and the Kruskal‐Wallis test for continuous characteristics. We used the Fine and Grey competing risk model to determine cumulative CBC risk, with death and non‐invasive CBCs as competing risks. The Cox proportional hazards model was used to estimate univariable and multivariable cause‐specific hazard ratios (HR) for CBC risk by histological PBC subtype; also the impact of neo‐adjuvant or adjuvant treatment was evaluated, which we defined as treatment with chemotherapy and/or endocrine therapy. Potential confounders added to the overall model were age at PBC, application of neo‐adjuvant or adjuvant systemic therapy (i.e., none, only chemotherapy, only endocrine therapy or both) and peri‐operative radiotherapy. Patients were followed from PBC diagnosis until the development of an invasive metachronous CBC and were censored at diagnosis of an ipsilateral invasive second breast tumour, a second invasive non‐breast tumour, occurrence of in situ CBC, death, or at last follow‐up (31/12/2015). We performed a subgroup analysis in PBC patients with the combination of positive oestrogen receptor (ER) status (irrespective of progesterone receptor [PR] status) or positive PR status (if ER status was unknown) and negative HER2‐status PBC, to estimate the HR for CBC in a more homogeneous patient population. Analyses were performed using Stata version 15.0.

To determine the cumulative incidence by CBC subtype, the other CBC subtypes were taken into account as competing events, and patients were censored at diagnosis of an ipsilateral second breast tumour, a second non‐breast tumour, non‐invasive CBC, death, or at last follow‐up (31/12/2015). R software (version 3.5.1) was used for this analysis.

Because metachronous CBC was defined as the development of a new PBC in the opposite breast at least 3 months after the PBC diagnosis, follow‐up started from 3 months onwards for all patients. Consequently, patients who developed an event within the first 3 months following PBC diagnosis were excluded (*n* = 1833).

The proportional hazards assumption was inspected using Schoenfeld residuals. Subsequently, we added interaction terms to investigate whether effect modification was present between the histologic groups and other variables included in the overall Cox model. In addition, we explored the presence of effect modification between the systemic therapy subgroups and the other variables in the stratified models within the histologic groups.

Five‐year follow‐up information on recurrent disease (local and distant) was available for all patients diagnosed with PBC between 2003 and 2006 (*n* = 35,512) and for 56% of the patients diagnosed between 2007–2008 (*n* = 11,103).

A sensitivity analysis was performed to investigate whether taking into account recurrent disease as a censoring event could lead to different results. For this analysis, only patients with complete 5‐year information on recurrent disease (i.e., local, regional and distant recurrence) were included. Censoring endpoints were diagnosis of recurrent disease, an ipsilateral invasive second breast tumour, a second invasive non‐breast tumour, occurrence of in situ CBC, death, or at last follow‐up (31/12/2015). The results were subsequently compared to the same group of patients ignoring recurrent disease as a censoring endpoint.

## 3 RESULTS

Clinical characteristics of the 74,373 included patients are presented in Table 1. During a median follow‐up time of 7.8 years, 2515 patients were diagnosed with a metachronous invasive CBC. Patients with lobular PBC were more often above 60 years of age at PBC diagnosis (54.0%) than the lobular mixed (45.4%) and the ductal (44.6%) group (*p* < 0.05). Patients with lobular mixed PBC were most often treated with both modalities (i.e., chemotherapy and endocrine therapy): 32% versus 35% versus 28% in lobular, lobular mixed and ductal PBC, respectively (*p* < 0.001), while the majority of the lobular and ductal PBC patients received no systemic treatment (33% vs. 35% vs. 37%, respectively).

**TABLE 1:** Characteristics of patients included comparing lobular vs. ductal and lobular mixed vs. ductal groups

	Total group		Lobular		Lobular mixed		Ductal		*p*‐value^a^
	*N*	*%*	*N*	*%*	*N*	*%*	*N*	*%*	
Total	***74,373***	100	***8903***	12.0	***3240***	4.4	***62,230***	83.7	
Median follow‐up in years [IQR]	7.8 [5.9–10.1]		7.8 [5.9–10.1]		8.2 [6.2–10.3]		7.8 [5.9–10.1]		<0.001^b^
Age at PBC									
Median age, years [range]	58.4 [19–101]		61.4 [20–98]		58.2 [21–95]		58.0 [19–101]		<0.05
<30	387	0.5	7	0.1	8	0.3	372	0.6	<0.001
30–39	4164	5.6	180	2.0	134	4.1	3850	6.2	
40–49	15,076	20.3	1612	18.1	667	20.6	12,797	20.6	
50–59	20,772	27.9	2331	26.2	957	29.5	17,484	28.1	
60–69	17,841	24.0	2307	25.9	766	23.6	14,768	23.7	
70–79	10,968	14.8	1642	18.4	512	15.8	8814	14.2	
80–89	4828	6.5	778	8.7	185	5.7	3865	6.2	
90+	337	0.5	46	0.5	11	0.3	280	0.5	
Stage									<0.001
IA	32,201	43.3	3157	35.5	1269	39.2	27,775	44.6	
IB	2943	4.0	233	2.6	119	3.7	2591	4.2	
IIA	19,191	25.8	2369	26.6	832	25.7	15,990	25.7	
IIB	9693	13.0	1408	15.8	469	14.5	7816	12.6	
IIIA	6255	8.4	994	11.2	342	10.6	4916	7.9	
IIIB	852	1.2	88	1.0	30	0.9	734	1.2	
IIIC	3238	4.4	654	7.4	179	5.5	2405	3.9	
Differentiation grade									<0.001
Grade I: well differentiated	14,655	21.3	1635	21.8	626	21.3	12,394	21.3	
Grade II: moderately differentiated	31,560	46.0	5056	67.3	1742	59.4	24,762	42.5	
Grade III: poorly differentiated/undifferentiated	22,456	32.7	827	11.0	566	19.3	21,063	36.2	
Unknown	5702		1385		306		4011		
Oestrogen receptor status									<0.001
Positive	58,521	82.2	8214	95.7	2909	94.1	47,398	79.6	
Negative	12,694	17.8	366	4.3	182	5.9	12,146	20.4	
Unknown/not determined	3158		323		149		2686		
Progesterone receptor status									<0.001
Positive	45,727	66.5	6288	76.6	2321	77.2	37,118	64.6	
Negative	22,997	33.5	1926	23.5	684	22.8	20,387	35.5	
Unknown/not determined	5649		689		235		4725		
HER2 receptor status									<0.001
Positive	8530	15.2	277	4.2	211	8.8	8042	17.1	
Negative	47,573	84.8	6400	95.9	2190	91.2	38,983	82.9	
Unknown/not determined	18,270		2226		839		15,205		
Surgery									<0.001
Lumpectomy	40,993	55.1	3638	40.9	1444	44.6	35,911	57.7	
Mastectomy	33,380	44.9	5265	59.1	1796	55.4	26,319	42.3	
Radiotherapy									<0.001
Yes	50,241	67.6	5486	61.6	1981	61.1	42,774	68.7	
No	24,132	32.5	3417	38.4	1259	38.9	19,456	31.3	
Chemotherapy									<0.005
Yes	31,417	42.2	3219	36.2	1312	40.5	26,886	43.2	
No	42,956	57.8	5684	63.8	1928	59.5	35,344	56.8	
Endocrine therapy									<0.001
Yes	37,047	49.8	5582	62.7	1949	60.2	29,516	47.4	
No	37,326	50.2	3321	37.3	1291	39.9	32,714	52.6	
Targeted therapy									<0.001
Yes	5181	7.0	137	1.5	119	3.7	4925	7.9	
No	69,192	93.0	8766	98.5	3121	96.3	57,305	92.1	
Systemic therapy									<0.001
No chemotherapy or endocrine therapy	27,180	36.6	2950	33.1	1120	34.6	23,110	37.1	
Only chemotherapy	10,146	13.6	371	4.2	171	5.3	9604	15.4	
Only endocrine therapy	15,776	21.2	2734	30.7	808	24.9	12,234	19.7	
Both chemotherapy and endocrine therapy	21,271	28.6	2848	32.0	1141	35.2	17,282	27.8	

Abbreviations: PBC, primary breast cancer; Stage: Stage I: T1N0M0 and T0‐1N1mi M0; Stage II: T0‐1N1M0, T2N0M0, T2N1M0, or T3N0M0; Stage III: T0‐2N2M0, T3N1‐2 M0, T4N0‐2 M0, or any T N3M0 breast cancer.

^a^ *p*‐values account for comparison lobular versus ductal and lobular mixed versus ductal PBC.

^b^ *p* = 0.7229 for lobular versus ductal.

### 3.1 CBC risk: Overall and by systemic treatment

**FIGURE 2:**
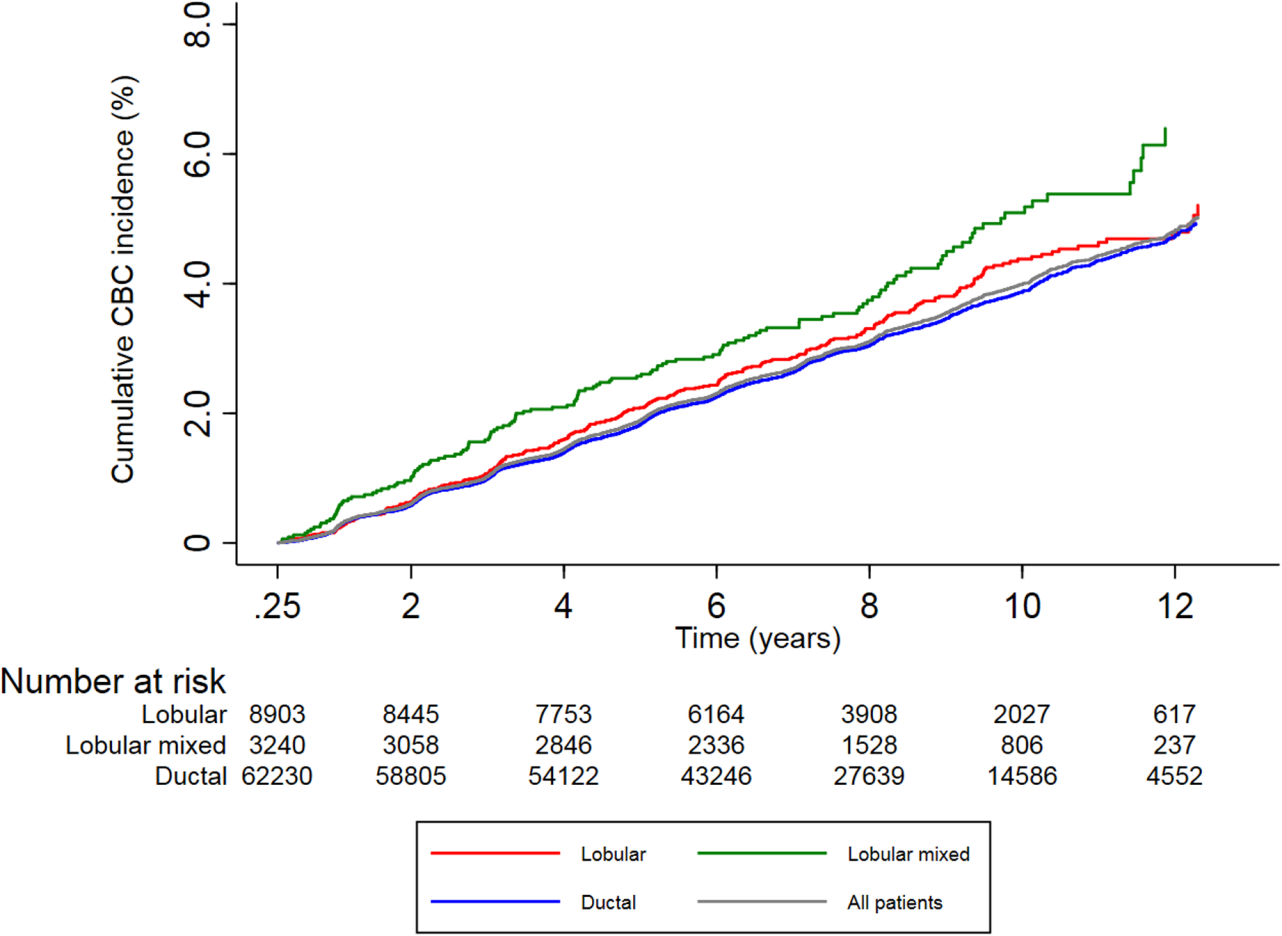
Cumulative incidence of developing metachronous invasive CBC according to PBC histology using competing risk analysis (%). CBC, contralateral breast cancer; PBC, primary breast cancer.

Ten‐year cumulative CBC incidence was 4.4% (95% CI: 3.9–4.9%) in the lobular group, 5.1% (95% CI: 4.3–6.0%) in the lobular mixed group versus 3.9% (95% CI: 3.7–4.0%) in the ductal group (Table 2; Figure 2).

**TABLE 2:** Five‐ and ten‐year cumulative metachronous CBC incidence according to histologic subtypes and systemic treatment, with death and non‐invasive CBC as competing risk

	All patients		Lobular		Lobular (mixed)		Ductal	
	*N* PBC	*N* CBC	*N* PBC	*N* CBC	*N* PBC	*N* CBC	*N* PBC	*N* CBC
Total	*74,373*	*2515*	*8903*	*319*	*3240*	*141*	*62,230*	*2055*
5‐year CBC risk % [95% CI]	**1.9** [1.8–2.0]		**2.1** [1.8–2.4]		**2.6** [2.1–3.2]		**1.8** [1.7–1.9]	
10‐year CBC risk % [95% CI]	**4.0** [3.8–4.2]		**4.4** [3.9–4.9]		**5.1** [4.3–6.0]		**3.9** [3.7–4.0]	
No systemic therapy received	*27,180*	*1393*	*2950*	*174*	*1120*	*80*	*23,110*	*1139*
5‐year CBC risk % [95% CI]	**3.0** [2.8–3.2]		**3.6** [2.9–4.3]		**3.8** [2.8–5.0]		**2.8** [2.6–3.1]	
10‐year CBC risk % [95% CI]	**5.8** [5.5–6.1]		**6.6** [5.6–7.6]		**7.7** [6.1–9.6]		**5.6** [5.2–5.9]	
Only chemotherapy received	*10,146*	*354*	*371*	*9*	*171*	*8*	*9604*	*337*
5‐year CBC risk % [95% CI]	**2.0** [1.7–2.3]		**2.2** [1.0–4.1]^a^		**3.6** [1.5–7.2]^c^		**1.9** [1.7–2.2]	
10‐year CBC risk % [95% CI]	**4.1** [3.7–4.6]		**NA** ^b^		**NA** ^d^		**4.2** [3.7–4.7]	
Only endocrine therapy received	*15,776*	*345*	*2734*	*61*	*808*	*24*	*12,234*	*260*
5‐year CBC risk % [95% CI]	**1.3** [1.1–1.5]		**1.3** [0.9–1.8]		**2.3** [1.4–3.5]		**1.2** [1.1–1.5]	
10‐year CBC risk % [95% CI]	**2.7** [2.4–3.0]		**3.0** [2.2–3.9]		**3.6** [2.3–5.3]^e^		**2.5** [2.2–2.9]	
Chemotherapy and endocrine therapy received	*21,271*	*423*	*2848*	*75*	*1141*	*29*	*17,282*	*319*
5‐year CBC risk % [95% CI]	**0.9** [0.8–1.1]		**1.3** [0.9–1.8]		**1.4** [0.9–2.3]		**0.8** [0.7–1.0]	
10‐year CBC risk % [95% CI]	**2.5** [2.2–2.8]		**3.5** [2.7–4.4]		**3.5** [2.3–5.1]		**2.3** [2.0–2.6]	

Abbreviations: NA, not applicable; *N* CBC, number of contralateral breast cancer events; *N* PBC, number of PBC patients. Time starts from 3 months after primary breast cancer diagnosis until metachronous CBC; *Censoring events*: ipsilateral recurrence including second breast cancer (either invasive or DCIS); second invasive non‐breast tumour (except non‐melanoma skin cancer); loss to follow‐up; end of follow‐up (31/12/2015); *competing events*: death and non‐invasive contralateral BC.

^a^ Time point of observation available was 4.5 years after PBC diagnosis.

^b^ Last time point of observation was at 7.5 years after PBC diagnosis.

^c^ Time point of observation available was 3.8 years after PBC diagnosis.

^d^ Only 2 events remained (i.e. at time points 6.6 and 11.4 years after PBC diagnosis).

^e^ Last time point of observation was at 8.9 years after PBC diagnosis.

In all patients who were treated with peri‐operative systemic therapy, that is, either chemotherapy and/or endocrine therapy, ten‐year cumulative CBC incidences were 3.2% (95% CI: 2.7–3.8%) and 3.6% (95% CI: 2.7–4.7%) versus 2.8% (95% CI: 2.6–3.0%) for the lobular, lobular mixed versus the ductal group, respectively. Ten‐year cumulative CBC incidences were 6.6% (95% CI: 5.6–7.6%) and 7.7% (95% CI: 6.1.7–9.6%) versus 5.6% (95% CI: 5.2–5.9%), respectively, if no systemic therapy was given.

There was no evidence for effect modification of CBC risk, not between the three histologic subgroups and the systemic therapy categories (Table 3), nor between the systemic therapy categories and the other variables included in the multivariable model stratified for the histologic subgroups (Table S1A,B).

**TABLE 3:** Univariable and multivariable Cox regression analyses for invasive metachronous CBC risk in the total group and the subset of patients with ER‐positive, HER2‐negative PBC

	*Total group*				*Subset analysis*			
	PYO	*N* CBC	uHR [95% CI]	mHR [95% CI]	PYO	*N* CBC	uHR [95% CI]	mHR [95% CI]
Total group	560,314	2515	—	—	288,186	1306	—	—
Lobular	66,754	319	1.09 [0.97–1.23]	1.18 [1.04–1.33]	43,255	199	1.04 [0.89–1.21]	1.12 [0.96–1.31]
Lobular (mixed)	24,866	141	1.29 [1.09–1.52]	1.37 [1.16–1.63]	15,117	87	1.29 [1.04–1.61]	1.36 [1.10–1.70]
Ductal	468,693	2055	Ref.	Ref.	229,813	1020	Ref.	Ref.
Systemic therapy								
None	212,195	1393	Ref.	Ref.	108,157	793	Ref.	Ref.
Only chemotherapy	71,773	354	0.76 [0.68–0.86]	0.76 [0.67–0.86]	6178	13	0.29 [0.17–0.50]	0.29 [0.17–0.51]
Only endocrine therapy	110,189	345	0.48 [0.43–0.55]	0.49 [0.43–0.55]	70,579	240	0.47 [0.41–0.54]	0.46 [0.39–0.53]
Both chemotherapy and endocrine therapy	166,157	423	0.39 [0.35–0.43]	0.38 [0.34–0.43]	103,272	260	0.35 [0.30–0.40]	0.35 [0.30–0.41]
Radiotherapy	385,424	1744	1.02 [0.94–1.11]	1.01 [0.92–1.10]	202,469	913	0.98 [0.87–1.10]	0.94 [0.84–1.06]
No Radiotherapy	174,889	771	Ref.	Ref.	85,717	393	Ref.	Ref.
Age (10‐year increase)	560,314	2515	1.02 [1.01–1.03]	0.98 [0.94–1.02]	288,186	1306	1.12 [1.07–1.17]	1.02 [0.97–1.07]

Abbreviations: ER, oestrogenestrogen receptor status; mHR, multivariable hazard ratio. *N* CBC, number of contralateral breast cancer events; PR, progesterone receptor status; PYO, person‐years of observation; uHR, univariable hazard ratio. Time starts from 3 months after primary breast cancer diagnosis until metachronous CBC; *Censoring events*: ipsilateral recurrence including second breast cancer (either invasive or in situ); non‐invasive contralateral breast cancer; second invasive non‐breast tumour (except non‐melanoma skin cancer); loss to follow‐up; end of follow‐up (31/12/2015); death.

The multivariable HR for CBC risk were increased for both the lobular (HR: 1.18, 95% CI: 1.04–1.33) and lobular mixed group (HR: 1.37, 95% CI: 1.16–1.63), as compared to ductal PBC patients (Table 3).

In the subset of patients with hormone receptor positive disease, 6170 lobular, 2094 lobular mixed and 32,393 ductal PBCs were included (of which 98.8% had ER+/HER2‐ PBC and 1.2% had ER unknown/PR+/HER2‐ PBC). Multivariable HR in this subset were similar to those in the total group, both for lobular as for lobular mixed compared to ductal PBC patients (HR: 1.12, 95% CI: 0.96–1.31, and HR: 1.36, 95% CI: 1.10–1.70, respectively; Table 3).

The lowest HR were seen for patients treated with both chemotherapy and endocrine therapy in all three subtypes (multivariable HR: 0.43, 95% CI: 0.32–0.57 for lobular, HR: 0.36, 95% CI: 0.23–0.56 for lobular mixed and HR: 0.37, 95% CI: 0.32–0.42 for ductal PBC patients; Table S1A). In the subselection of hormone receptor positive PBC patients, HR were 0.39 (95% CI: 0.27–0.57), 0.35 (95% CI: 0.20–0.62) and 0.34 (95% CI: 0.28–0.40), respectively (Table S1B).

We observed no substantial alterations in the sensitivity analysis when censoring for recurrent disease versus ignoring recurrent disease; negligible differences were observed between the multivariable HR (Table S3).

### 3.2 Comparisons of CBC characteristics

**FIGURE 3:**
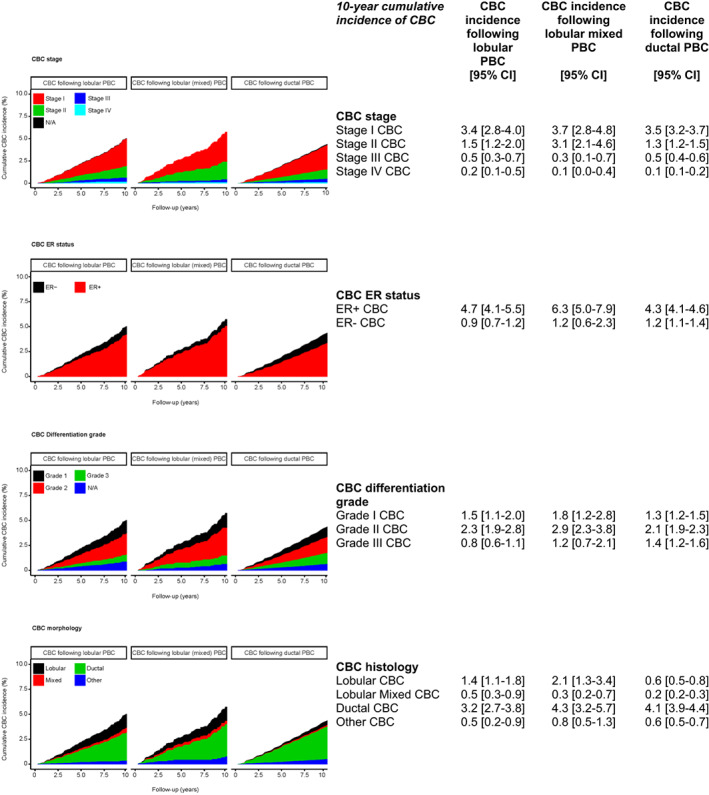
stacked 10‐year cumulative metachronous invasive CBC subtype incidence according to PBC histology (%). 95% CI, 95% Confidence Interval; CBC, contralateral breast cancer; ER, Oestrogen receptor status; PBC, primary breast cancer. Stage: Stage I: T1N0M0 and T0‐1N1mi M0; Stage II: T0‐1N1M0, T2N0M0, T2N1M0, or T3N0M0; Stage III: T0‐2N2M0, T3N1‐2 M0, T4N0‐2 M0, or any T N3M0 breast cancer; stage IV: metastatic breast cancer.

The cumulative CBC subtype incidence curves are shown in Figure 3, separately for patients with a lobular, lobular mixed or ductal PBC. The majority of CBCs had favourable tumour characteristics (i.e. stage I, ER‐positive, grade I/II), with a similar distribution between the three groups.

### 3.3 CBC versus PBC characteristics

In 59 (19%), 36 (26%) and 470 (23%) of the lobular, lobular mixed and ductal PBC patients who developed a CBC, respectively, a more advanced stage than in the primary tumour was observed (Table S2A‐C). The CBC differentiation grade was higher than the PBC differentiation grade in 52 (22%), 38 (33%) and 459 (27%) patients, respectively.

Further, all PBC patients mainly developed ductal CBCs (179 (56%), 78 (55%) and 1541 (75.0%), respectively). Patients with lobular (36%) or lobular mixed PBC (26%) more often developed a lobular/lobular mixed CBC than in ductal patients (14.3%).

## 4 DISCUSSION

In this nationwide cohort study with over 74,000 patients, we observed that lobular and lobular mixed type PBC was associated with a higher risk of CBC than ductal PBC. The application of neo‐adjuvant or adjuvant systemic therapy was associated with a decreased CBC risk among patients, irrespective of PBC histology. Further, in about a quarter of the CBC patients with a lobular mixed or ductal PBC, and in approximately a fifth of CBC patients with lobular PBC, the characteristics of the CBCs were worse, i.e., higher stage and grade, than their primary tumour.

The lobular mixed type PBCs tended to be associated with the highest CBC risk increase (HR: 1.37 compared to ductal), which is in line with the study published by Peiro et al.^10^ They investigated lobular mixed with ductal histology subtypes. The majority of the mixed types in our dataset concerned lobular mixed with ductal histology subtypes as well (92%); for the remaining 8%, we had no information on the accompanying subtypes.

We also found an increased risk of CBC for patients with lobular PBC in comparison to ductal PBC. This is in line with multiple other studies,^2^, ^3^, ^4^, ^6^, ^7^, ^11^ although in the older studies (mainly studies published prior to 2000), the effect sizes were larger, with relative risks ranging from 1.7–2.0. The attenuation might be explained by the fact that in recent studies the more extensive use of peri‐operative systemic therapy resulted in a decreased risk association, as has been shown for CBC risk in general.^12^ Since the introduction of adjuvant endocrine therapy for ER‐positive BC, lobular PBCs, in which ER is expressed more frequently than in ductal PBCs, have probably been treated with endocrine treatment more often. We therefore assume that absolute CBC risk decreased more in lobular compared to in ductal PBCs, resulting in a lower relative CBC risk. Indeed in our study, ER‐positivity was observed in 96% of lobular and 94% of lobular mixed and 80% in ductal PBC, respectively. Patients with lobular and lobular‐mixed PBCs were treated with endocrine therapy more often than patients with ductal PBCs in our study (65%, 64% and 59%, respectively, when restricted to ER‐positive BC patients; Table 1).

Within the group of lobular PBC patients, the associations of chemotherapy with CBC risk were comparable to the associations of endocrine therapy alone and of endocrine therapy in combination with chemotherapy. However, the observational nature of our study and the small number of events in the chemotherapy group within lobular PBC patients prohibit us from drawing strong conclusions on the impact of different systemic therapy types on CBC risk.

The histology of the PBC and the CBC was more often similar in patients with lobular or lobular mixed PBCs than in patients with ductal PBC. The similarity in histology between the primary and secondary breast cancer might suggest that part of these tumours could be a metastatic spread of the primary tumour, rather than a new entity. From literature it is known that lobular breast cancers have a diffuse growth pattern and metastasize more often, perhaps also affecting the contralateral breast.^6^, ^13^, ^14^ Since lobular BCs have been difficult to visualise on mammography,^15^ these metastatic spreads might have been missed initially and may have been classified as new primary tumours later on. Genetic analysis investigating clonality between a primary and second primary tumour may shed light on CBC being a true primary tumour or a metastatic disease in these cases. Lifestyle factors (e.g. hormone replacement therapy use) and germline pathogenic variants such as in the *CDH1* gene are also associated with the development of multiple lobular breast cancers.^16^, ^17^, ^18^

This study used a large and comprehensive population‐based dataset to evaluate metachronous CBC risk in lobular and lobular‐mixed PBC patients. In addition, complete information on second breast cancer occurrence was present. This makes our results generalizable, although a limitation to our study is that we did not have complete follow‐up information concerning recurrent disease for patients diagnosed between 2007 and 2010. Especially the occurrence of metastatic disease might be of importance, because in that case, patients will be mainly treated with systemic therapy, potentially lowering their risk of CBC. The sensitivity analysis in the group with complete information on recurrent disease which was censored for at occurrence confirmed our initial findings, suggesting negligible bias. Further research is needed though, especially in ER‐positive BCs, which have a tendency to develop metastases after longer time periods than 5 years following PBC diagnosis.

Another potential limitation of our study is the lack of information on the presence of germline pathogenic mutations. This could have resulted in an overestimation of CBC risk in the present study. However, no association of lobular histology with known breast cancer gene mutations (*BRCA1/2*, *CHEK2*, *PALB2* and *ATM*) has been reported in literature so far.^17^ Therefore, we do not expect that this will be a confounding factor in our study.

In conclusion, lobular and lobular‐mixed histology of PBC are associated with increased risks of CBC as compared to ductal PBC. Personalised CBC risk assessment needs to consider PBC histology, including the administration of peri‐operative systemic treatment. The impact on prognosis of CBCs with unfavourable characteristics warrants further evaluation.

## FUNDING INFORMATION

This work was supported by the Dutch Cancer Society/Alpe d'HuZes [A6C/6253].

## CONFLICT OF INTEREST

The authors have declared no conflicts of interest.

## ETHICAL APPROVAL STATEMENT

The review boards of the Netherlands Cancer Registry and the Dutch nationwide network and registry of histopathology and cytopathology (PALGA) approved the proposal and the data were handled in accordance to the privacy regulations for medical research (Federa_code_of_conduct_english.pdf [bbmri.nl]).

## Supporting information


Tables S1‐S3

## Data Availability

The data that support the findings of this study are available from the Netherlands Cancer Registry. Restrictions apply to the availability of these data, which were used under licence for this study. Data are available at www.iknl.nl with the permission of the Netherlands Comprehensive Cancer Organisation.
