# Supplementary material for: Risk of metachronous contralateral breast cancer in patients with primary invasive lobular breast cancer: Results from a nationwide cohort
Source: Cancer Med. 2022 Sep 20;12(3):3123–33. doi: 10.1002/cam4.5235 (PMC9939202; doi:10.1002/cam4.5235)
Supplement: Supplementary file 1 — Tables S1‐S3 [file CAM4-12-3123-s001.docx]

**Supplementary Material**

**Supplementary Table S1A:** univariable and multivariable Cox regression analyses of invasive metachronous CBC risk stratified by histologic subtype
**Supplementary Table S1B:** univariable and multivariable Cox regression analyses of invasive metachronous CBC risk for ER/PR-positive with HER2- negative PBC stratified by histologic subtype

**Supplementary Table S2A:** metachronous CBC characteristics of women with lobular PBC

**Supplementary Table S2B:** metachronous CBC characteristics of women with lobular mixed PBC

**Supplementary Table S2C:** metachronous CBC characteristics of women with ductal PBC

***Sensitivity analysis****To investigate whether ignoring recurrent disease in the main analysis could lead to biased results, we performed a sensitivity analysis including only patients with complete 5-year information on recurrent (local/regional/distant) disease. For this analysis, 46,591 patients were included*

**Supplementary Table S3:** sensitivity analysis: univariable and multivariable Cox regression analyses for invasive metachronous CBC risk in patients with complete follow-up on recurrent disease

| **Supplementary Table S1A: univariable and multivariable Cox regression analyses of invasive metachronous CBC risk stratified by histologic subtype** | | | | | | | | | | |
| --- | --- | --- | --- | --- | --- | --- | --- | --- | --- | --- |
|  | ***Lobular*** | | | ***Lobular mixed*** | | | | ***Ductal*** | | |
|  | **PYO (*N* CBC)** | **uHR [95% CI]** | **mHR  [95% CI]** | **PYO (*N* CBC)** | | **uHR [95% CI]** | **mHR  [95% CI]** | **PYO (*N* CBC)** | **uHR [95% CI]** | **mHR [95% CI]** |
| **Total group** | 66,754 (319) | - | - | 24,866 (141) | - | | - | 468,693 (2,055) | - | - |
|  |  |  |  |  |  | |  |  |  |  |
| **Systemic therapy** |  |  |  |  |  | |  |  |  |  |
| No chemotherapy or endocrine therapy | 23,682 (174) | **Ref.** | **Ref.** | 9,131 (80) | **Ref.** | | **Ref.** | 179,382  (1,139) | **Ref.** | **Ref.** |
| Only chemotherapy | 2,557 (9) | 0.49 [0.25-0.95] | 0.45 [0.23-0.88] | 1,183 (8) | 0.78 [0.38-1.61] | | 0.73 [0.35-1.54] | 68,033 (337) | 0.73 [0.64-0.82] | 0.78 [0.69-0.89] |
| Only endocrine therapy | 18,852 (61) | 0.45 [0.33-0.60] | 0.48 [0.36-0.65] | 5,697 (24) | 0.49 [0.31-0.77] | | 0.51 [0.32-0.82] | 85,639 (260) | 0.45 [0.39-0.51] | 0.49 [0.42-0.56] |
| Both chemotherapy and endocrine therapy | 21,664 (75) | 0.47 [0.36-0.62] | 0.43 [0.32-0.57] | 8,856 (29) | 0.38 [0.25-0.58] | | 0.36 [0.23-0.56] | 135,638 (319) | 0.38 [0.34-0.43] | 0.37 [0.32-0.42] |
|  |  |  |  |  |  | |  |  |  |  |
| **Radiotherapy** | 41,582  (209) | 1.15 [0.91-1.45] | 1.09 [0.86-1.38] | 15,312 (92) | 1.17 [0.83-1.65] | | 1.16 [0.81-1.64] | 328,530 (1,443) | 1.07 [0.98-1.18] | 0.98 [0.89-1.08] |
| No radiotherapy | 25,172 (110) | **Ref.** | **Ref.** | 9,554 (49) | **Ref.** | | **Ref.** | 140,163 (612) | **Ref.** | **Ref.** |
|  |  |  |  |  |  | |  |  |  |  |
| **Age** (10-year increase) | 66,754 (319) | 0.9 [0.94-1.02] | 0.91 [0.82-1.01] | 24,866 (141) | 1.01 [0.96-1.08] | | 0.95 [0.81-1.12] | 468,693 (2,055) | 1.00 [0.997-1.003] | 0.99 [0.95-1.03] |
|  |  |  |  |  |  | |  |  |  |  |
| Abbreviations: PYO=Person-years of observation; *N* *CBC*=Number of contralateral breast cancer events; *uHR*=univariable hazard ratio; *mHR*=multivariable hazard ratio.  Estimates in the multivariable model were adjusted for all variables from the univariable model.  Time starts from 3 months after primary breast cancer diagnosis until metachronous contralateral breast cancer; *Censoring events*: ipsilateral recurrence including second breast cancer (either invasive or in situ); in situ contralateral breast cancer; second invasive non-breast tumour (except non-melanoma skin cancer); loss to follow-up; end of follow-up (31/12/2015); death | | | | | | | | | | |

| **Supplementary Table S1B: univariable and multivariable Cox regression analyses of invasive metachronous CBC risk for ER/PR-positive with HER2- negative PBC stratified by histologic subtype** | | | | | | | | | | |
| --- | --- | --- | --- | --- | --- | --- | --- | --- | --- | --- |
|  | ***Lobular*** | | | ***Lobular mixed*** | | | | ***Ductal*** | | |
|  | **PYO (*N* CBC)** | **uHR [95% CI]** | **mHR  [95% CI]** | **PYO (*N* CBC)** | | **uHR [95% CI]** | **mHR  [95% CI]** | **PYO (*N* CBC)** | **uHR [95% CI]** | **mHR [95% CI]** |
| **Total group** | 43,255 (199) | - | - | 15,117 (87) | - | | - | 229,813 (1,020) | - | - |
|  |  |  |  |  |  | |  |  |  |  |
| **Systemic therapy** |  |  |  |  |  | |  |  |  |  |
| No chemotherapy or endocrine therapy | 13,073 (99) | **Ref.** | **Ref.** | 5,041 (48) | **Ref.** | | **Ref.** | 90,043  (646) | **Ref.** | **Ref.** |
| Only chemotherapy | 1,008 (2) | 0.26 [0.06-1.06] | 0.24 [0.06-0.99] | 313 (0) | * | | * | 4,857 (11) | 0.32 [0.18-0.58] | 0.33 [0.18-0.60] |
| Only endocrine therapy | 13,003 (46) | 0.47 [0.33-0.67] | 0.50 [0.35-0.71] | 3,735 (19) | 0.54 [0.32-0.93] | | 0.55 [0.31-0.95] | 53,840 (175) | 0.46 [0.39-0.54] | 0.44 [0.37-0.53] |
| Both chemotherapy and endocrine therapy | 16,171 (52) | 0.43 [0.30-0.60] | 0.39 [0.27-0.57] | 6,029 (20) | 0.35 [0.21-0.60] | | 0.35 [0.20-0.62] | 81,073 (188) | 0.32 [0.28-0.38] | 0.34 [0.28-0.40] |
|  |  |  |  |  |  | |  |  |  |  |
| **Radiotherapy** | 27,343  (130) | 1.09 [0.81-1.46] | 1.04 [0.77-1.40] | 9,345 (55) | 1.06 [0.69-1.65] | | 1.06 [0.68-1.64] | 165,781 (728) | 0.95 [0.83-1.09] | 0.91 [0.79-1.04] |
| No radiotherapy | 15,912 (69) | **Ref.** | **Ref.** | 5,773 (32) | **Ref.** | | **Ref.** | 64,032 (292) | **Ref.** | **Ref.** |
|  |  |  |  |  |  | |  |  |  |  |
| **Age** (10-year increase) | 43,255 (199) | 1.01 [0.90-1.13] | 0.93 [0.80-1.07] | 15,117 (87) | 1.13 [0.94-1.35] | | 1.00 [0.82-1.24] | 229,813 (1,020) | 1.14 [1.08-1.20] | 1.04 [0.98-1.10] |
|  |  |  |  |  |  | |  |  |  |  |
| Abbreviations: ER=estrogen receptor status; PR=progesterone receptor status; PYO=person-years of observation; *N* *CBC*=number of contralateral breast cancer events; *uHR*=univariable hazard ratio; *mHR*=multivariable hazard ratio.  Estimates in the multivariable model were adjusted for all variables from the univariable model.  Time starts from 3 months after primary breast cancer diagnosis until metachronous contralateral breast cancer; *Censoring events*: ipsilateral recurrence including second breast cancer (either invasive or in situ); in situ contralateral breast cancer; second invasive non-breast tumour (except non-melanoma skin cancer); loss to follow-up; end of follow-up (31/12/2015); death | | | | | | | | | | |

**Supplementary Tables S2A-C:**

Metachronous invasive CBC characteristics of women with different histologic PBC subtypes.

Abbreviations: *CBC*=contralateral breast cancer; *PB*C=primary breast cancer; ER=Oestrogen receptor status;
PR=Progesterone receptor status

Stage**:** Stage I: T1N0M0 and T0-1N1mi M0; Stage II: T0-1N1M0, T2N0M0, T2N1M0, or T3N0M0; Stage III: T0-2N2M0, T3N1-2M0, T4N0-2M0, or any T N3M0 breast cancer; stage IV: metastatic breast cancer

**Supplementary Table S2A: metachronous CBC characteristics of women with lobular PBC**

| **CBC characteristics** |  | ***PBC characteristics*** | | | | | | | | | | | |  | *Total* | | | | |  |
| --- | --- | --- | --- | --- | --- | --- | --- | --- | --- | --- | --- | --- | --- | --- | --- | --- | --- | --- | --- | --- |
|  |  | ***N*** | | ***%*** | | | | | ***N*** | ***%*** | | ***N*** | ***%*** |  | ***N*** | ***%*** | | | | ***p*-value** |
|  |  | **PBC histology** | | | | | | | | | | | | |  | | |  | | |
| **CBC histology** |  | Lobular | | | | | | |  | | | | |  |  | | | |  | |
| Lobular |  | 89 | *27.9* | | | | | |  | | |  | |  |  | | | |  | |
| Lobular mixed |  | 26 | *8.2* | | | | | |  | | |  | |  |  | | | |  | |
| Ductal |  | 179 | *56.1* | | | | | |  | | |  | |  |  | | | |  | |
| Other |  | 25 | *7.8* | | | | | |  | | |  | |  |  | | | |  | |
| *Total* |  | *319* | *100* | | | | | |  | | |  | |  |  | | | |  | |
|  |  |  | | | | | | | | | |  | |  |  | | | |  | |
|  |  | **PBC TNM-stage** | | | | | | | | | | | |  |  | | | |  | |
|  |  | I | | | | | | | II | | | III | |  |  | | | |  | |
| **CBC TNM-stage** |  |  | | | | | | | | | | | |  |  | | | | <0.001 | |
| I |  | 113 | | *71.5* | | | | | 59 | *58.4* | | 18 | *32.1* |  | 190 | *60.3* | | |  | |
| II |  | 34 | | *21.5* | | | | | 35 | *34.7* | | 15 | *26.8* |  | 84 | *26.7* | | |  | |
| III |  | 9 | | *5.7* | | | | | 4 | *4.0* | | 16 | *28.6* |  | 29 | *9.2* | | |  | |
| IV |  | 2 | | *1.3* | | | | | 3 | *3.0* | | 7 | *12.5* |  | 12 | *3.8* | | |  | |
| *Total* |  | *158* | | *100* | | | | | *101* | *100* | | *56* | *100* |  | *315* | *100* | | |  | |
|  |  |  | | | | | | | | | | | |  |  | | | |  | |
|  |  | **PBC differentiation grade** | | | | | | | | | | | |  |  | | | |  | |
|  |  | I | | | | | | | II | | | III | |  |  | | | |  | |
| **CBC differentiation grade** |  |  | | | | | | | | | | | |  |  | | | | 0.007 | |
| I |  | 26 | | | *52.0* | | | | 40 | *25.2* | | 5 | *21.7* |  | 71 | *30.6* | | |  | |
| II |  | 18 | | | *36.0* | | | | 91 | *57.2* | | 13 | *56.5* |  | 122 | *52.6* | | |  | |
| III |  | 6 | | | *12.0* | | | | 28 | *17.6* | | 5 | *21.7* |  | 39 | *16.8* | | |  | |
| *Total* |  | 50 | | | *100* | | | | 159 | *100* | | 23 | *100* |  | 232 | *100* | | |  | |
|  |  |  | | | | | | |  | | |  | |  |  | | | |  | |
|  |  | **PBC ER status** | | | | | | | | | | | |  |  | | | |  | |
|  |  | Positive | | | | | | | Negative | | |  | |  |  | | | |  | |
| **CBC ER status** |  |  | | | | | | |  | | |  | |  |  | | | | 0.169 | |
| Positive |  | 249 | | | | *86.2* | | | 11 | *73.3* | |  | |  | 260 | *85.5* | | |  | |
| Negative |  | 40 | | | | *13.8* | | | 4 | *26.7* | |  | |  | 44 | *14.5* | | |  | |
| *Total* |  | 289 | | | | *100* | | | 15 | *100* | |  | |  | 304 | *100* | | |  | |
|  |  |  | | | | | | | | | |  | |  |  | | | |  | |
|  |  | **PBC PR status** | | | | | | | | | |  | |  |  | | | |  | |
|  |  | Positive | | | | | | Negative | | |  | | |  |  | | | |  | |
| **CBC PR status** |  |  | | | | | |  | | |  | | |  |  | | | |  | |
| Positive |  | 142 | | | | | *62.0* | 30 | | *54.6* |  | | |  | 172 | | *60.6* | |  | |
| Negative |  | 87 | | | | | *38.0* | 25 | | *45.5* |  | | |  | 112 | | *39.4* | |  | |
| *Total* |  | 229 | | | | | *100* | 55 | | *100* |  | | |  | 284 | | *100* | |  | |
|  |  |  | | | | | | | |  |  | | |  |  | | | |  | |
|  |  | **PBC HER2 status** | | | | | | | | |  | | | |  | | |  | | |
|  |  | Positive | | | | | | Negative | | |  | | |  |  | | | |  | |
| **CBC HER2 status** |  |  | | | | | |  | | |  | | |  |  | | | | 0.015 | |
| Positive |  | 4 | | | | | *26.7* | 15 | | *7.8* |  | | |  | 19 | | *9.2* | |  | |
| Negative |  | 11 | | | | | *73.3* | 177 | | *92.2* |  | | |  | 188 | | *90.8* | |  | |
| *Total* |  | 15 | | | | | *100* | 192 | | *100* |  | | |  | 207 | | *100* | |  | |

| **CBC characteristics** |  | ***PBC characteristics*** | | | | | | | | | | | |  | *Total* | | | | |  |
| --- | --- | --- | --- | --- | --- | --- | --- | --- | --- | --- | --- | --- | --- | --- | --- | --- | --- | --- | --- | --- |
|  |  | ***N*** | | ***%*** | | | | | ***N*** | ***%*** | | ***N*** | ***%*** |  | ***N*** | ***%*** | | | | ***p*-value** |
|  |  | **PBC histology** | | | | | | | | | | | | |  | | |  | | |
| **CBC histology** |  | Lobular mixed | | | | | | |  | | | | |  |  | | | |  | |
| Lobular |  | 36 | *25.5* | | | | | |  | | |  | |  |  | | | |  | |
| Lobular mixed |  | 10 | *7.1* | | | | | |  | | |  | |  |  | | | |  | |
| Ductal |  | 78 | *55.3* | | | | | |  | | |  | |  |  | | | |  | |
| Other |  | 17 | *12.1* | | | | | |  | | |  | |  |  | | | |  | |
| *Total* |  | *141* | *100* | | | | | |  | | |  | |  |  | | | |  | |
|  |  |  | | | | | | | | | |  | |  |  | | | |  | |
|  |  | **PBC TNM-stage** | | | | | | | | | | | |  |  | | | |  | |
|  |  | I | | | | | | | II | | | III | |  |  | | | |  | |
| **CBC TNM-stage** |  |  | | | | | | | | | | | |  |  | | | | 0.004 | |
| I |  | 46 | | *59.0* | | | | | 24 | *57.1* | | 8 | *40.0* |  | 78 | *55.7* | | |  | |
| II |  | 30 | | *38.5* | | | | | 16 | *38.1* | | 6 | *30.0* |  | 52 | *37.1* | | |  | |
| III |  | 2 | | *2.6* | | | | | 1 | *2.4* | | 4 | *20.0* |  | 7 | *5.0* | | |  | |
| IV |  | 0 | | *0* | | | | | 1 | *2.4* | | 2 | *10.0* |  | 3 | *2.1* | | |  | |
| *Total* |  | *78* | | *100* | | | | | *42* | *100* | | *20* | *100* |  | *140* | *100* | | |  | |
|  |  |  | | | | | | | | | | | |  |  | | | |  | |
|  |  | **PBC differentiation grade** | | | | | | | | | | | |  |  | | | |  | |
|  |  | I | | | | | | | II | | | III | |  |  | | | |  | |
| **CBC differentiation grade** |  |  | | | | | | | | | | | |  |  | | | | 0.161 | |
| I |  | 6 | | | *19.4* | | | | 24 | *35.8* | | 3 | *16.7* |  | 33 | *28.4* | | |  | |
| II |  | 21 | | | *67.7* | | | | 30 | *44.8* | | 10 | *55.6* |  | 61 | *52.6* | | |  | |
| III |  | 4 | | | *12.9* | | | | 13 | *19.4* | | 5 | *27.8* |  | 22 | *19.0* | | |  | |
| *Total* |  | 31 | | | *100* | | | | 67 | *100* | | 18 | *100* |  | 116 | *100* | | |  | |
|  |  |  | | | | | | |  | | |  | |  |  | | | |  | |
|  |  | **PBC ER status** | | | | | | | | | | | |  |  | | | |  | |
|  |  | Positive | | | | | | | Negative | | |  | |  |  | | | |  | |
| **CBC ER status** |  |  | | | | | | |  | | |  | |  |  | | | | 0.236 | |
| Positive |  | 112 | | | | *90.3* | | | 7 | *77.8* | |  | |  | 119 | *89.5* | | |  | |
| Negative |  | 12 | | | | *9.7* | | | 2 | *22.2* | |  | |  | 14 | *10.5* | | |  | |
| *Total* |  | 124 | | | | *100* | | | 9 | *100* | |  | |  | 133 | *100* | | |  | |
|  |  |  | | | | | | | | | |  | |  |  | | | |  | |
|  |  | **PBC PR status** | | | | | | | | | |  | |  |  | | | |  | |
|  |  | Positive | | | | | | Negative | | |  | | |  |  | | | |  | |
| **CBC PR status** |  |  | | | | | |  | | |  | | |  |  | | | | 0.335 | |
| Positive |  | 65 | | | | | *66.3* | 17 | | *56.7* |  | | |  | 82 | | *64.1* | |  | |
| Negative |  | 33 | | | | | *33.7* | 13 | | *43.3* |  | | |  | 46 | | *35.9* | |  | |
| *Total* |  | 98 | | | | | *100* | 30 | | *100* |  | | |  | 128 | | *100* | |  | |
|  |  |  | | | | | | | |  |  | | |  |  | | | |  | |
|  |  | **PBC HER2 status** | | | | | | | | |  | | | |  | | |  | | |
|  |  | Positive | | | | | | Negative | | |  | | |  |  | | | |  | |
| **CBC HER2 status** |  |  | | | | | |  | | |  | | |  |  | | | | 0.291 | |
| Positive |  | 2 | | | | | *28.6* | 12 | | *13.8* |  | | |  | 14 | | *14.9* | |  | |
| Negative |  | 5 | | | | | *71.4* | 75 | | *86.2* |  | | |  | 80 | | *85.1* | |  | |
| *Total* |  | 7 | | | | | *100* | 87 | | *100* |  | | |  | 94 | | *100* | |  | |

**Supplementary Table S2B: metachronous CBC characteristics of women with lobular mixed PBC**

**Supplementary Table S2C: metachronous CBC characteristics of women with ductal PBC**

| **CBC characteristics** |  | ***PBC characteristics*** | | | | | | | | | | | |  | *Total* | | | | |  |
| --- | --- | --- | --- | --- | --- | --- | --- | --- | --- | --- | --- | --- | --- | --- | --- | --- | --- | --- | --- | --- |
|  |  | ***N*** | | ***%*** | | | | | ***N*** | ***%*** | | ***N*** | ***%*** |  | ***N*** | ***%*** | | | | ***p*-value** |
|  |  | **PBC histology** | | | | | | | | | | | | |  | | |  | | |
| **CBC histology** |  | Ductal | | | | | | |  | | | | |  |  | | | |  | |
| Lobular |  | 217 | *10.6* | | | | | |  | | |  | |  |  | | | |  | |
| Lobular mixed |  | 75 | *3.7* | | | | | |  | | |  | |  |  | | | |  | |
| Ductal |  | 1,541 | *75.0* | | | | | |  | | |  | |  |  | | | |  | |
| Other |  | 222 | *10.8* | | | | | |  | | |  | |  |  | | | |  | |
| *Total* |  | *2,055* | *100* | | | | | |  | | |  | |  |  | | | |  | |
|  |  |  | | | | | | | | | |  | |  |  | | | |  | |
|  |  | **PBC TNM-stage** | | | | | | | | | | | |  |  | | | |  | |
|  |  | I | | | | | | | II | | | III | |  |  | | | |  | |
| **CBC TNM-stage** |  |  | | | | | | | | | | | |  |  | | | | <0.001 | |
| I |  | 861 | | *69.7* | | | | | 316 | *56.7* | | 101 | *47.9* |  | 1,278 | *63.8* | | |  | |
| II |  | 280 | | *22.7* | | | | | 164 | *29.4* | | 48 | *22.8* |  | 420 | *21.0* | | |  | |
| III |  | 76 | | *6.2* | | | | | 60 | *10.8* | | 44 | *20.9* |  | 180 | *9.0* | | |  | |
| IV |  | 19 | | *1.5* | | | | | 17 | *3.1* | | 18 | *8.5* |  | 54 | *2.7* | | |  | |
| *Total* |  | *1,236* | | *100* | | | | | *557* | *100* | | *211* | *100* |  | *2,004* | *100* | | |  | |
|  |  |  | | | | | | | | | | | |  |  | | | |  | |
|  |  | **PBC differentiation grade** | | | | | | | | | | | |  |  | | | |  | |
|  |  | I | | | | | | | II | | | III | |  |  | | | |  | |
| **CBC differentiation grade** |  |  | | | | | | | | | | | |  |  | | | | <0.001 | |
| I |  | 182 | | | *39.7* | | | | 202 | *27.2* | | 81 | *17.0* |  | 465 | *27.7* | | |  | |
| II |  | 201 | | | *43.9* | | | | 357 | *48.1* | | 171 | *35.8* |  | 729 | *43.4* | | |  | |
| III |  | 75 | | | *16.4* | | | | 183 | *24.7* | | 226 | *47.3* |  | 484 | *28.8* | | |  | |
| *Total* |  | 458 | | | *100* | | | | 742 | *100* | | 478 | *100* |  | 1,678 | *100* | | |  | |
|  |  |  | | | | | | |  | | |  | |  |  | | | |  | |
|  |  | **PBC ER status** | | | | | | | | | | | |  |  | | | |  | |
|  |  | Positive | | | | | | | Negative | | |  | |  |  | | | |  | |
| **CBC ER status** |  |  | | | | | | |  | | |  | |  |  | | | | <0.001 | |
| Positive |  | 1,264 | | | | *86.8* | | | 239 | *55.6* | |  | |  | 1,503 | *79.7* | | |  | |
| Negative |  | 192 | | | | *13.2* | | | 191 | *44.4* | |  | |  | 383 | *20.3* | | |  | |
| *Total* |  | 1,456 | | | | *100* | | | 430 | *100* | |  | |  | 1,886 | *100* | | |  | |
|  |  |  | | | | | | | | | |  | |  |  | | | |  | |
|  |  | **PBC PR status** | | | | | | | | | |  | |  |  | | | |  | |
|  |  | Positive | | | | | | Negative | | |  | | |  |  | | | |  | |
| **CBC PR status** |  |  | | | | | |  | | |  | | |  |  | | | | <0.001 | |
| Positive |  | 761 | | | | | *65.9* | 294 | | *46.5* |  | | |  | 1,055 | | *59.0* | |  | |
| Negative |  | 394 | | | | | *34.1* | 338 | | *53.5* |  | | |  | 732 | | *41.0* | |  | |
| *Total* |  | 1,155 | | | | | *100* | 632 | | *100* |  | | |  | 1,787 | | *100* | |  | |
|  |  |  | | | | | | | |  |  | | |  |  | | | |  | |
|  |  | **PBC HER2 status** | | | | | | | | |  | | | |  | | |  | | |
|  |  | Positive | | | | | | Negative | | |  | | |  |  | | | |  | |
| **CBC HER2 status** |  |  | | | | | |  | | |  | | |  |  | | | | <0.001 | |
| Positive |  | 53 | | | | | *30.6* | 105 | | *8.7* |  | | |  | 158 | | *11.4* | |  | |
| Negative |  | 120 | | | | | *69.4* | 1,103 | | *91.3* |  | | |  | 1,223 | | *88.6* | |  | |
| *Total* |  | 173 | | | | | *100* | 1,208 | | *100* |  | | |  | 1,381 | | *100* | |  | |

***Sensitivity analysis***

To investigate whether ignoring recurrent disease in the main analysis could lead to biased results, we performed a sensitivity analysis.

Five-year follow-up on recurrences was complete for patients diagnosed with PBC between 2003 and 2006 and for 56% of the patients diagnosed between 2007-2008. For patients diagnosed between 2009-2010 no information on recurrences was available. With the subgroup of patients with complete 5-year follow-up information on recurrent disease, we performed a sensitivity analysis in order to evaluate the impact of taking into account recurrent disease on the results. For this analysis, local, regional and distance recurrences were also considered as a censoring endpoint, next to censoring at diagnosis of an ipsilateral second breast tumour, a second non-breast tumour, non-invasive CBC, death, or last follow-up (31/12/2015).

The analyses were compared to the same subset with ignoring local, regional and distant recurrence as an endpoint.

In total, 46, 591 patients with complete 5-year follow-up information on recurrent disease were included for this analysis. HRs from the sensitivity analysis were overlapping with those from the primary analysis.

| **Supplementary Table S3: sensitivity analysis: univariable and multivariable Cox regression analyses for invasive metachronous CBC risk in patients with complete follow-up on recurrent disease** | | | | | | | | |
| --- | --- | --- | --- | --- | --- | --- | --- | --- |
|  | **PYO** | | ***N* CBC** | | **uHR [95% CI]** | | **mHR [95% CI]** | |
| **Total group** | 207,681 | 214,921 | 874 | 934 | - | - | - | - |
| **Lobular** | 25,242 | 25,992 | 126 | 133 | 1.24 [1.02-1.50] | 1.23 [1.02-1.48] | 1.35 [1.12-1.64] | 1.36 [1.13-1.64] |
| Lobular (mixed) | 9,918 | 10,205 | 52 | 56 | 1.30 [0.98-1.72] | 1.32 [1.00-1.73] | 1.40 [1.05-1.85] | 1.43 [1.09-1.88] |
| Ductal | 172,617 | 178,724 | 696 | 745 | **Ref.** | **Ref.** | **Ref.** | **Ref.** |
|  |  |  |  |  |  |  |  |  |
| **Systemic therapy** |  |  |  |  |  |  |  |  |
| No chemotherapy or endocrine therapy | 84,404 | 86,683 | 534 | 546 | **Ref.** | **Ref.** | **Ref.** | **Ref.** |
| Only chemotherapy | 25,130 | 26,780 | 121 | 144 | 0.77 [0.63-0.94] | 0.86 [0.72-1.03] | 0.79 [0.64-0.98] | 0.87 [0.72-1.06] |
| Only endocrine therapy | 40,960 | 42,139 | 108 | 113 | 0.42 [0.34-0.52] | 0.43 [0.35-0.52] | 0.41 [0.33-0.51] | 0.42 [0.34-0.52] |
| Both chemotherapy and endocrine therapy | 57,284 | 59,319 | 111 | 131 | 0.31 [0.25-0.37] | 0.35 [0.29-0.42] | 0.30 [0.24-0.37] | 0.34 [0.27-0.41] |
|  |  |  |  |  |  |  |  |  |
| **Radiotherapy** | 142,209 | 146,861 | 594 | 637 | 0.97 [0.84-1.12] | 0.99 [0.86-1.14] | 0.96 [0.83-1.11] | 0.98 [0.85-1.12] |
| No Radiotherapy | 65,569 | 68,060 | 280 | 297 | **Ref.** | **Ref.** | **Ref.** | **Ref.** |
|  |  |  |  |  |  |  |  |  |
| **Age** (10-year increase) | 207,681 | 214,921 | 874 | 934 | 1.03 [1.00-1.05] | 1.03 [0.98-1.09] | 0.99 [0.93-1.05] | 0.98 [0.92-1.04] |
|  |  |  |  |  |  |  |  |  |
| Abbreviations: PYO=Person-years of observation; *N CBC*=number of contralateral breast cancer events; *uHR*=univariable hazard ratio; *mHR*=multivariable hazard ratio. Estimates in the multivariable model were adjusted for all variables from the univariable model.  Time starts from 3 months after primary breast cancer diagnosis until metachronous contralateral breast cancer; *Censoring events*: local, regional or distant recurrence, ipsilateral second breast cancer (either invasive or in situ); in situ contralateral breast cancer; second invasive non-breast tumour (except non-melanoma skin cancer); loss to follow-up; end of follow-up (31/12/2015); death. Hazard ratios are given for the first 5 years since information on recurrence is only available for the first 5 years of follow-up.  Numbers in red depict the outcome when local, regional and metastatic disease was ignored as a censoring event. | | | | | | | | |
